# Predictive value of soluble CD59 for poor 28-day neurological prognosis and all-cause mortality in patients after cardiopulmonary resuscitation: a prospective observatory study

**DOI:** 10.1186/s40560-023-00653-8

**Published:** 2023-02-02

**Authors:** Ling Wang, Rui-Fang Li, Xiao-Lan Guan, Shuang-Shuang Liang, Ping Gong

**Affiliations:** 1 Department of Neurology, The Affiliated Jinyang Hospital of Guizhou Medical University, Guiyang, Guizhou China; 2 Department of Emergency, First Affiliated Hospital of Dalian Medical University, Dalian, Liaoning China; 3 Department of Emergency, General Hospital of Tianjin Medical University, Tianjin, China; 4 Department of Emergency, Shenzhen People’s Hospital (The Second Clinical Medical College, Jinan University, The First Affiliated Hospital, Southern University of Science and Technology), Shenzhen, Guangdong Province China

**Keywords:** Cardiopulmonary resuscitation, Ischemia–reperfusion injury, Complement, Soluble CD59, Prognosis

## Abstract

**Background:**

sCD59, as a soluble form of CD59, is observed in multiple types of body fluids and correlated with the cell damage after ischemia/reperfusion injury. This study aims to observe the dynamic changes of serum sCD59 in patients after restoration of spontaneous circulation (ROSC) and explore the association of serum sCD59 with neurological prognosis and all-cause mortality in patients after ROSC.

**Methods:**

A total of 68 patients after ROSC were prospectively recruited and divided into survivors (*n* = 23) and non-survivors (*n* = 45) groups on the basis of 28-day survival. Twenty healthy volunteers were enrolled as controls. Serum sCD59 and other serum complement components, including sC5b-9, C5a, C3a, C3b, C1q, MBL, Bb, and pro-inflammatory mediators tumor necrosis factor (TNF)-α, interleukin-6 (IL-6), neurological damage biomarkers neuron-specific enolase (NSE) and soluble protein 100β (S100β) were measured by enzyme linked immunosorbent assay on day 1, 3, and 7 after ROSC. Neurologic outcome was assessed using cerebral performance category scores, with poor neurologic outcome defined as 3–5 points.

**Results:**

In the first week after ROSC, serum levels of sCD59, sC5b-9, C5a, C3a, C3b, C1q, MBL, Bb, TNF-α, IL-6, NSE and S100β were significantly elevated in patients after ROSC compared to healthy volunteers, with a significant elevation in the non-survivors compared to survivors except serum C1q and MBL. Serum sCD59 levels were positively correlated with serum sC5b-9, TNF-α, IL-6, NSE, S100β, SOFA score and APACHE II score. Moreover, serum sCD59 on day 1, 3, and 7 after ROSC could be used for predicting poor 28-day neurological prognosis and all-cause mortality. Serum sCD59 on day 3 had highest AUCs for predicting poor 28-day neurological prognosis [0.862 (95% CI 0.678–0.960)] and 28-day all-cause mortality [0.891 (95% CI 0.769–0.962)]. In multivariate logistic regression analysis, the serum level of sCD59_D1_ was independently associated with poor 28-day neurological prognosis and all-cause mortality.

**Conclusions:**

The elevated serum level of sCD59 was positively correlated with disease severity after ROSC. Moreover, serum sCD59 could have good predictive values for the poor 28-day neurological prognosis and all-cause mortality in patients after ROSC.

**Supplementary Information:**

The online version contains supplementary material available at 10.1186/s40560-023-00653-8.

## Background

Cardiac arrest (CA) remains one of the leading causes of disability and death worldwide despite great advances in the public training of cardiopulmonary resuscitation (CPR) and emergency and critical care medicine [1]. A substantial proportion of CA deaths still occur in patients following successful resuscitation due to long time hypoxia and a whole-body ischemia/reperfusion (I/R) injury [2]. Many survivors also have different degrees of neurologic impairments because brain is the most susceptible to the I/R injury [3]. The pathophysiological process of I/R injury after restoration of spontaneous circulation (ROSC) is very complex, and known as post-cardiac arrest syndrome (PACS) [2]. Especially, systemic inflammation is a hallmark of the post-cardiac arrest syndrome, together with the release of inflammatory mediators and the activation of complement system, which is closely correlated with neurological disability and high mortality [4, 5].

The complement system, as an important part of the innate immune system, mainly comprises the classical, lectin and alternative pathways [6]. Previous studies revealed that the activation of complement system was closely associated with ischemia/reperfusion injury [6–10]. Our previous animal study indicated that complement was activated through classical, lectin and alternative pathways after ROSC [11]. In addition, this activation of complement may be related with the initiation and development of the systemic inflammatory response, which synergistically contributes to post-resuscitation I/R injury [11]. During the complement activation, the membrane attack complex (MAC, namely C5b-9), is ultimately formed as a terminal product of the complement cascade [12]. The C5b-9 can attack cytomembrane directly, and is also transformed into a soluble molecule (sC5b-9) [12]. Circulating sC5b-9 has been observed to be elevated in many clinical settings, including sepsis, burn injury, transplantation, and trauma, which leads to the secretion of pro-inflammatory mediators and thus correlates with prognosis and neurological disability [12–17]. Thus, the complement inhibitor CD59 that blocks the assembly of C5b-9 through prohibiting the coupling of C9 to C5b-8, may be involved in inflammatory response and neurological dysfunction [18, 19].

CD59, as a glycophosphoinositol (GPI)-anchored protein expressed ubiquitously on cells, can protect cells from MAC-mediated osmotic cell lysis [20]. CD59 can also be released in vesicles or shed from cell surfaces into the circulation to form soluble CD59 (sCD59) [20]. Previous studies have reported the pathogenic role of MAC and the protective role of CD59 in hepatic, renal, gastric and myocardial I/R injury [21–23]. Additionally, sCD59 has been revealed to be capable of alleviating inflammation and liver damage in animal model of trichloroethylene induced immune liver injury [24]. However, until now the association of sCD59 with systemic inflammation and brain injury and the role of sCD59 in assessing the neurological outcome and mortality in patients after ROSC remain unclear. Thus, here we hypothesized serum sCD59 may alleviate systemic inflammation and brain injury and be used as a useful biomarker for early evaluation of the neurological outcome and all-cause mortality in patients after ROSC.

## Methods

### Ethical approval of the study protocol

This study was carried out in accordance with the Declaration of Helsinki (2013 edition) adopted by the World Medical Association [25]. The protocol was approved by the Medical Ethics Committee of the First Affiliated Hospital of Dalian Medical University (PJ-KS-KY-2019-150). Written informed consent was obtained from the relatives of all patients upon their initial admission to the hospital or from healthy volunteers.

### Study population

This prospective study was conducted in the emergency intensive care unit (ICU) and cardiac ICU in the First Affiliated Hospital of Dalian Medical University (Dalian, China). Patients after ROSC were enrolled from January 1, 2017 to October 30, 2019. All enrolled patients received intensive care management such as mechanical ventilation, sedation, hemodynamic support, fever prevention and treatment, seizure treatment, glucose control upon ICU admission according to the 2015 International Consensus on Cardiopulmonary Resuscitation [26]. All patients were divided into two groups (survivors and non-survivors) on the basis of survival or death at day 28 after ROSC. Concurrently, sex- and age-matched healthy volunteers were enrolled as a control group.

### Inclusion and exclusion criteria

**Fig. 1:**
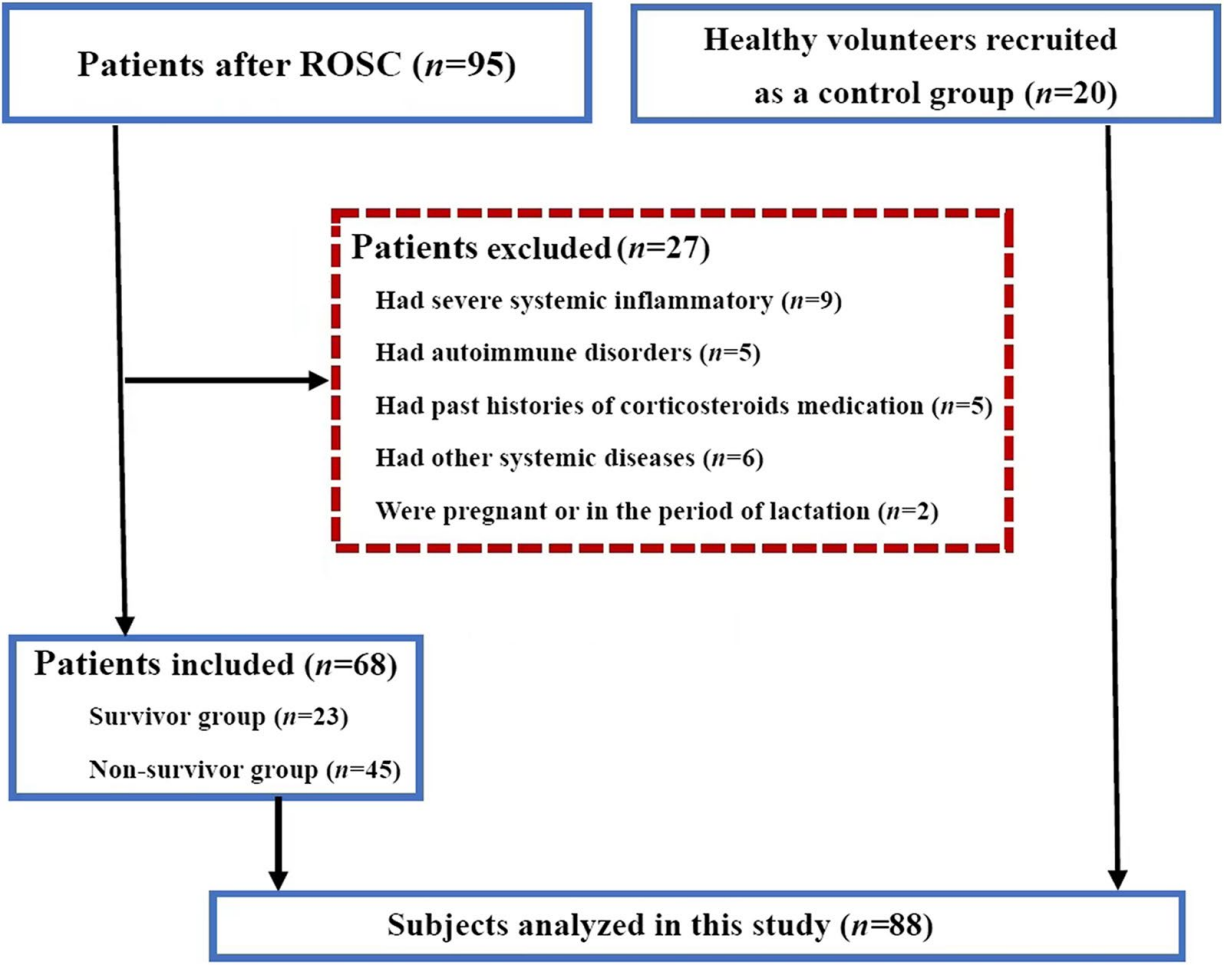
Flowchart of study participants. ROSC restoration of spontaneous circulation

Patients aged over 18 years old and resuscitated from Out-of-Hospital CA or In-Hospital CA who survived to 2 h or longer were included. Patients were excluded when they had sepsis, severe burns and trauma, major surgery, severe acute pancreatitis, or autoimmune diseases (including systemic lupus erythematosus, vasculitis, rheumatoid arthritis, scleroderma, Sjögren's syndrome, inflammatory myopathies) upon hospital admission, past histories of corticosteroids medication and other systemic diseases such as hematological diseases and malignancies, and were pregnant or in the period of lactation (Fig. 1).

### Clinical data collection

The patients’ demographic data and clinical parameters were prospectively collected from the medical records including baseline demographic data, medical history, causes of CA, initial heart rhythm, CPR time, laboratory findings and outcomes. The Sequential Organ Failure Assessment (SOFA) score and Acute Physiology and Chronic Health Evaluation (APACHE II) score were calculated on day 1, 3, and 7 after ROSC on the basis of age, medical history, vital signs, and laboratory results. Neurologic outcome was assessed using cerebral performance category (CPC) scores that were recorded on day 28 after ROSC, with a good neurologic outcome defined as 1–2 points and a poor neurologic outcome defined as 3–5 points.

### Blood sampling protocol

Venous blood samples were collected, respectively, from the patients at 2 h, 72 h and 168 h after ROSC or healthy volunteers on enrolled day. We have collected the blood samples at 2 h of all patients who survived to 1 day or longer, the blood samples at 72 h only in patients who survived to 3 days or longer after ROSC, and the blood samples at 168 h only in patients who survived to 7 days or longer after ROSC. Sample collectors, clinical investigators, assistants, and laboratory personnel were unaware of the study protocol. Blood samples were centrifuged (2500×*g*) at 4 °C for 10 min to collect the serum. The serum samples were aliquoted and stored at − 80 °C until further analysis. Enzyme-linked immunosorbent assay (ELISA) kits were used to measure serum sCD59 (Elabscience, Wuhan, China), sC5b-9 (Elabscience, Wuhan, China), C1q (Elabscience, Wuhan, China), MBL (CUSABIO, Wuhan, China), Bb (Quidel, California, USA), C3a (CUSABIO, Wuhan, China), C3b (CUSABIO, Wuhan, China), C5a (CUSABIO, Wuhan, China), IL-6 (Elabscience, Wuhan, China), TNF-a (Elabscience, Wuhan, China), NSE (CUSABIO, Wuhan, China) and S100β (CUSABIO, Wuhan, China) in accordance with the manufactures' instructions.

### Statistical analysis

All data were analyzed and visualized by SPSS v25.0 (IBM, Armonk, NY, USA) and GraphPad Prism 8 (GraphPad Software Inc., La Jolla, CA, USA). Through systematic evaluation of sample size, 17 patients in survivor group, 17 patients in non-survivor group and 17 healthy volunteers would provide a statistical power of 90% with a two-sided α = 0.05 to detect a 0.5 to 5.0 difference in three time points (day 1, 3, and 7 after ROSC) among three groups (survivor group, non-survivor group, and healthy volunteers) for the change in sCD59 (a main variable in the present study) [27]. The normality of continuous variables was assessed by the Kolmogorov–Smirnov test or Shapiro–Wilk test. The continuous variables were described as mean ± standard deviation (SD) or median (interquartile range) according to the normality, while categorical variables were described as counts (percentage). The categorical variables were compared by Pearson Chi-squared or Fisher exact tests. Repeated-measure analysis of variance (ANOVA) or Kruskal–Wallis one-way ANOVA was used to compare the changes of variables at different time points among the survivors, non-survivors and healthy volunteers, followed by Bonferroni tests for multiple comparisons or Mann–Whitney *U* test for two-group comparisons. The association between sCD59 levels and other parameters was assessed by Spearman’s correlation. To determine whether sCD59 could be independent predictors to poor 28-day neurological prognosis or 28-day all-cause mortality after ROSC, binary logistic regression analyses were performed, and the results were presented as odds ratio (OR) and 95% confidence interval (CI). To investigate the associations between sCD59 levels and poor 28-day neurological prognosis or 28-day all-cause mortality, receiver operating characteristic (ROC) curves were generated and the areas under the ROC curves (AUCs) were calculated and compared by DeLong's test. After determining the optimal thresholds through analyzing the ROC curves, prognostic parameters (sensitivity, specificity, positive predictive value [PPV], negative predictive value [NPV], Youden Index, positive likelihood ratio [LR+] and negative likelihood ratio [LR−]) were also calculated. Statistical differences were considered significantly if *P* < 0.05.

## Results

### Baseline characteristics of the study participants

In this study, we collected 20 (29.4%) patients with in-hospital cardiac arrest (IHCA) and 48 (70.6%) patients with out-of-hospital cardiac arrest (OHCA), and enrolled 20 healthy volunteers (Table 1). Twenty-three patients survived to 28 days after ROSC, and 45 patients died of instability or re-arrest (*n* = 12), brain death (*n* = 11), multiple organ dysfunction (*n* = 9), refractory cardiogenic shock (*n* = 8), respiratory failure (*n* = 3) and heart failure (*n* = 2). Accordingly, the number of cases in the survivors and non-survivors on day 1, day 3, and day 7 were 23 and 45, 23 and 26, 23 and 18, respectively (Fig. 1 and Additional file 1: Table S1). The duration from ROSC to death were 2.0 (1.0, 5.0) days for patients died in the first week after ROSC and 5.0 (2.0, 13.0) days for all patients died in the 28 days after ROSC. No significant differences were presented in age and sex among healthy volunteers, survivors and non-survivors (all *P* > 0.05, Tables 1 and Additional file 1: Table S1). The causes of CA, comorbidities and main treatments (mechanical ventilation, sedation, hemodynamic support, seizure treatment) were not significantly different between non-survivors and survivors. All the patients after ROSC received conventional fever prevention rather than therapeutic hypothermia during the study because the standardized target temperature management devices were not available in our ICUs. However, the non-survivors had shorter duration of ICU-stay when compared to the survivors (all *P* < 0.05, Table 1 and Additional file 1: Table S1). There were significant increases in CPR time, SOFA score and APACHE II score in the non-survivors compared to the survivors (all *P* < 0.05, Table 1 and Additional file 1: Table S1). A total of 60.8% (14/23) survivors had a 28-day CPC score of 1 to 2 points, indicating a good neurological prognosis.

**Table 1:** Baseline characteristics of the study cohort

	Healthy volunteers	Survivors	Non-survivors
	(*n* = 20)	(*n* = 23)	(*n* = 45)
Age (years)	58.4 ± 13.6	58.5 ± 16.1	65.0 ± 15.3
Male sex [*n* (%)]	12 (60.0%)	17 (73.9%)	28 (62.2%)
Comorbidities [*n* (%)]			
Hypertension	–	9 (39.1%)	22 (48.9%)
Diabetes	–	5 (21.8%)	12 (26.7%)
Coronary heart disease	–	11 (47.8%)	18 (40.0%)
Chronic pulmonary disease	–	3 (13.0%)	4 (8.8%)
Chronic renal disease	–	2 (8.7%)	5 (11.1%)
Episode location [*n* (%)]			
OHCA	–	14 (60.9%)	34 (75.6%)
IHCA	–	9 (39.1%)	11 (24.4%)
Causes of cardiac arrest [*n* (%)]			
Cardiac	–	11 (47.8%)	20 (44.4%)
Respiratory	–	4 (17.4%)	10 (22.2%)
Cerebral	–	3 (13.1%)	8 (17.8%)
Others	–	5 (21.7%)	7 (15.6%)
Causes of death [*n* (%)]			
Instability or re-arrest	–	–	12 (26.7%)
Brain death	–	–	11 (24.4%)
Multiple organ dysfunction	–	–	9 (20.0%)
Refractory cardiogenic shock	–	–	8 (17.7%)
Respiratory failure	–	–	3 (6.7%)
Heart failure	–	–	2 (4.5%)
Initial cardiac rhythm [*n* (%)]			
Shockable rhythm	–	14 (60.9%)	18 (40.0%)
Non-shockable rhythm	–	9 (39.1%)	27 (60.0%)
CPR time (min)	–	7.0 (3.0, 10.0)	15.0 (8.0, 23.0)^a^
Length of ICU-stay (days)	–	10.0 (6.0, 17.0)	5.0 (2.0, 11.5)^a^
Treatments [*n* (%)]			
Ventilator use	–	23 (100%)	45 (100%)
Sedation	–	23 (100%)	45 (100%)
Hemodynamic support	–	20 (87.0%)	45 (100%)
Seizure treatment	–	4 (17.4%)	9 (20.0%)
Laboratory findings			
Leukocyte count (× 10^9^/L)	6.66 (5.55, 8.45)	12.46 (8.34, 19.32)^b^	12.99 (9.81, 22.37)^b^
PCT (ng/mL)	0.06 (0.03, 0.09)	1.36 (0.81, 1.74)^b^	2.00 (1.00, 3.47)^b^
hs-troponin I (μg/L)	0.01 (0.00, 0.02)	0.10 (0.06, 1.70)^b^	0.87 (0.38, 6.80)^b^
BNP (pg/mL)	26.5 (17.8, 37.8)	124.0 (49.0, 370.9)^b^	659.0 (183.0, 1994.7)^a^
Lactate (mmol/L)	0.50 (0.25, 1.29)	2.00 (1.40, 4.70)^b^	6.40 (2.60, 11.70)^a^
Creatinine (μmol/L)	70.4 (61.8, 76.9)	83.0 (60.0, 106.0)^b^	149.0 (100.3, 243.5)^a^
APACHE II scores	–	20.0 (14.0, 38.0)	38.0 (34.0, 41.5)^a^
SOFA scores	–	5.0 (4.0, 7.5)	10.0 (9.0, 13.0)^a^

Values are the mean ± standard deviation, median (interquartile range) or *n* (percentile)

*APACHE II* Acute Physiology and Chronic Health Evaluation II, *BNP* brain natriuretic peptide, *CPC* cerebral performance category, *CPR* cardiopulmonary resuscitation, *hs-troponin I* high-sensitivity troponin I, *ICU* intensive care unit, *IHCA* in-hospital cardiac arrest, *OHCA* out-of-hospital cardiac arrest, *PCT* procalcitonin, *SOFA* Sequential Organ Failure Assessment

^a^*P* < 0.05 vs survivors, ^b^*P* < 0.05 vs healthy volunteers

Compared to healthy volunteers, patients after ROSC had increased levels of leukocyte count, procalcitonin (PCT), high-sensitivity troponin I (hs-TnI), brain natriuretic peptide (BNP), lactate and creatinine at baseline (all *P* < 0.05, Table 1). Baseline levels of blood leukocyte count, PCT and hs-TnI were not different in the survivors and non-survivors; whereas compared to the survivors, BNP, lactate and creatinine levels at baseline were significantly higher in the non-survivors (all *P* < 0.05, Table 1).

### Comparisons of serum C1q, Bb, MBL, C3b, C3a, C5a, sC5b-9 and sCD59 levels among healthy volunteers, survivors and non-survivors

**Fig. 2:**
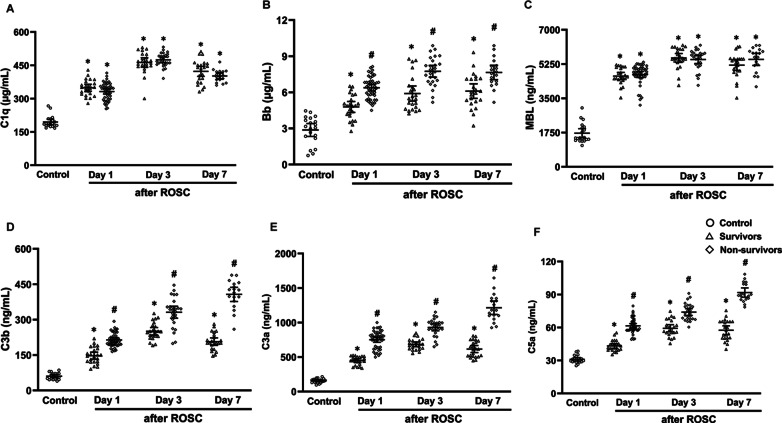
Comparison of serum levels of C1q (**A**), Bb (**B**), MBL (**C**), C3b (**D**), C3a (**E**) and C5a (**F**) in control (healthy volunteers), survivors, and non-survivors. MBL mannose binding lectin, ROSC restoration of spontaneous circulation. ^*^P < 0.05 versus Healthy volunteers; ^#^*P* < 0.05 versus Survivors

**Fig. 3:**
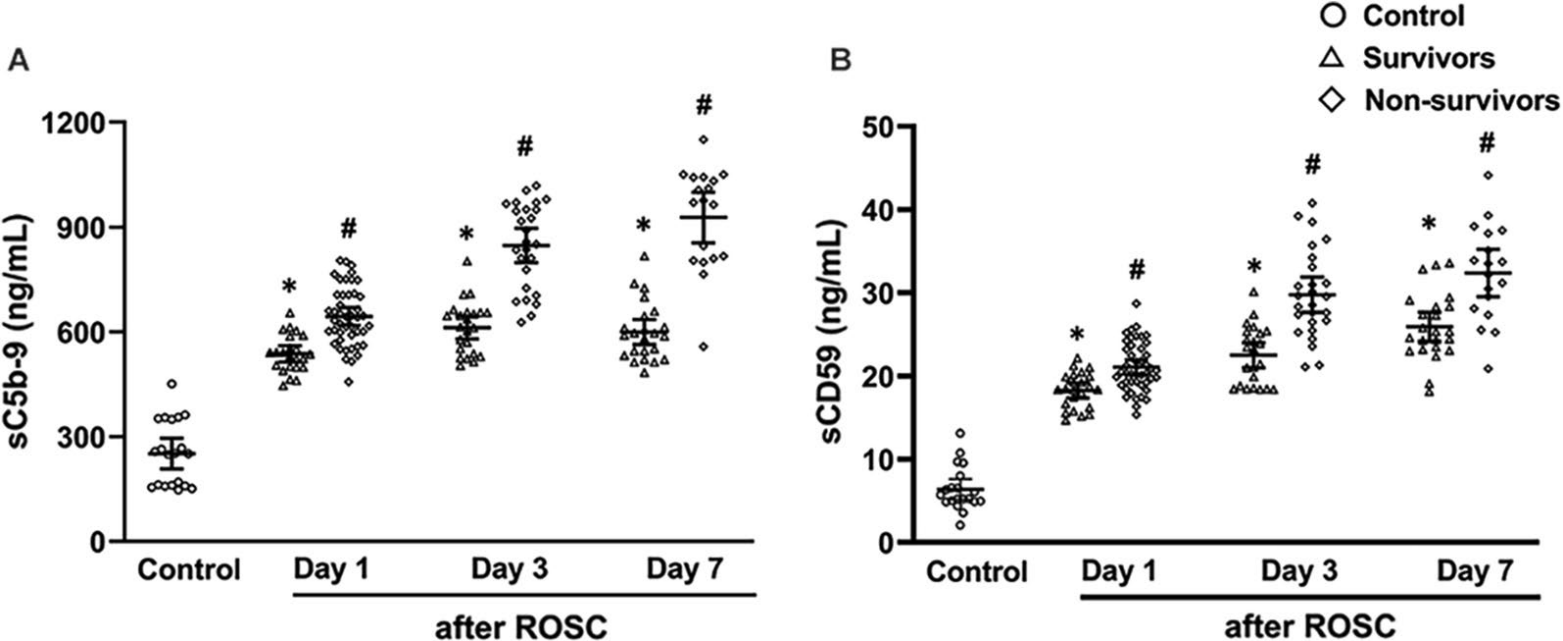
Comparison of serum levels of sC5b-9 (**A**) and sCD59 (**B**) in control (healthy volunteers), survivors, and nonsurvivors. ROSC restoration of spontaneous circulation, sC5b-9 soluble complement terminal complex, sCD59 soluble CD59. ^*^P < 0.05 versus Healthy volunteers; ^#^*P* < 0.05 versus Survivors

**Fig. 4:**
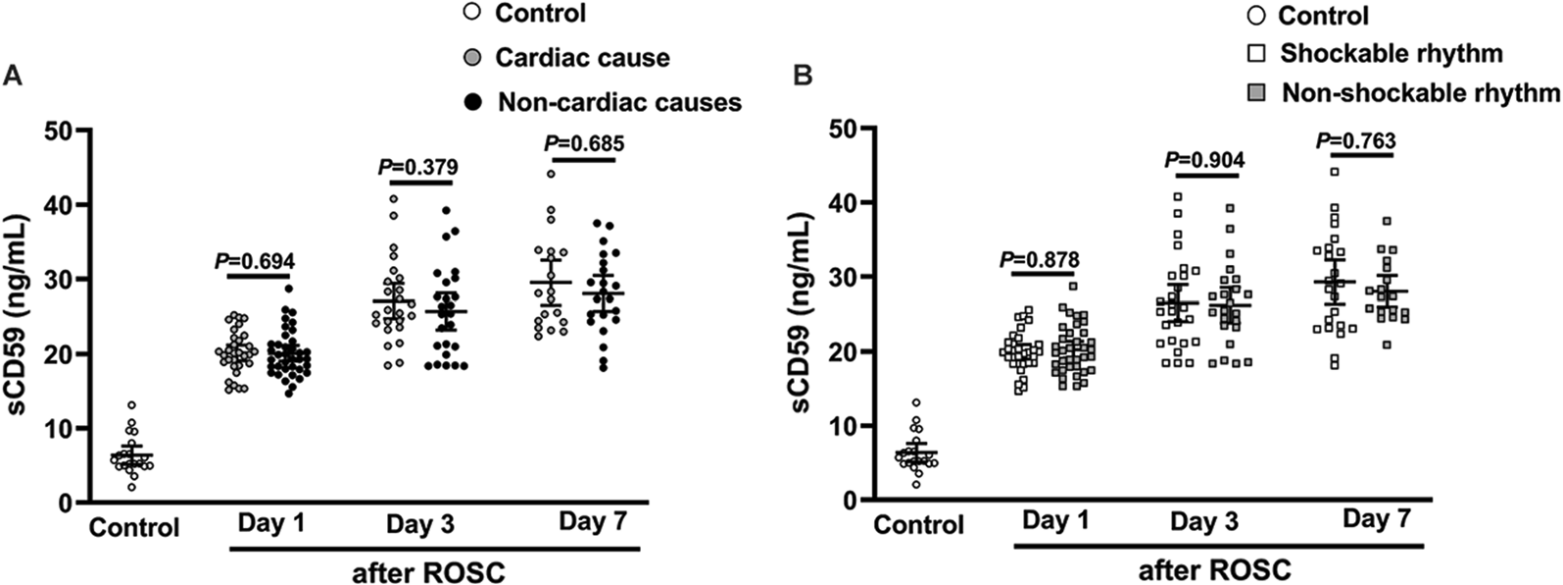
Comparison of serum levels of sCD59 among control (healthy volunteers), cardiac arrest patients with cardiac cause or non-cardiac causes (**A**), and among control (healthy volunteers), cardiac arrest patients with shockable rhythm or non-shockable rhythm (**B**). sCD59 soluble CD59

In the first week after ROSC, serum levels of C1q, Bb, MBL, C3b, C3a, C5a, sC5b-9 and sCD59 were significantly elevated in patients after ROSC compared to healthy volunteers (all *P* < 0.05, Figs. 2 and 3). Notably, serum level of sCD59 was gradually elevated from day 1 to day 7 after ROSC in either survivors or non-survivors (all *P* < 0.05, Additional file 1: Table S2). Serum levels of C1q and MBL did not differ between the non-survivors and survivors, whereas serum levels of remaining complements were all significantly increased in the non-survivors compared to the survivors (all *P* < 0.05, Figs. 2 and 3). Furthermore, serum levels of sCD59 in early death patients (died within the first 7 days after ROSC) on day 1 and 3 after ROSC were significantly higher than those in survivors (all *P* < 0.05, Additional file 1: Table S3). There were no significant differences in serum sCD59 between patients with cardiac cause and non-cardiac causes and between patients with the initial cardiac rhythm of shockable rhythm and non-shockable rhythm in either survivors or non-survivors on day 1, 3 and 7 after ROSC (all *P* > 0.05; Fig. 4, Additional file 1: Tables S4 and S5). In addition, no significant difference in AUCs of sCD59 was found in patients with cardiac/non-cardiac causes and shockable rhythm/non-shockable on day 1, 3 and 7 after ROSC (all *P* > 0.05, Additional file 1: Table S6).

### Comparisons of serum TNF-α, IL-6, NSE and S100β levels among healthy volunteers, survivors and non-survivors

**Fig. 5:**
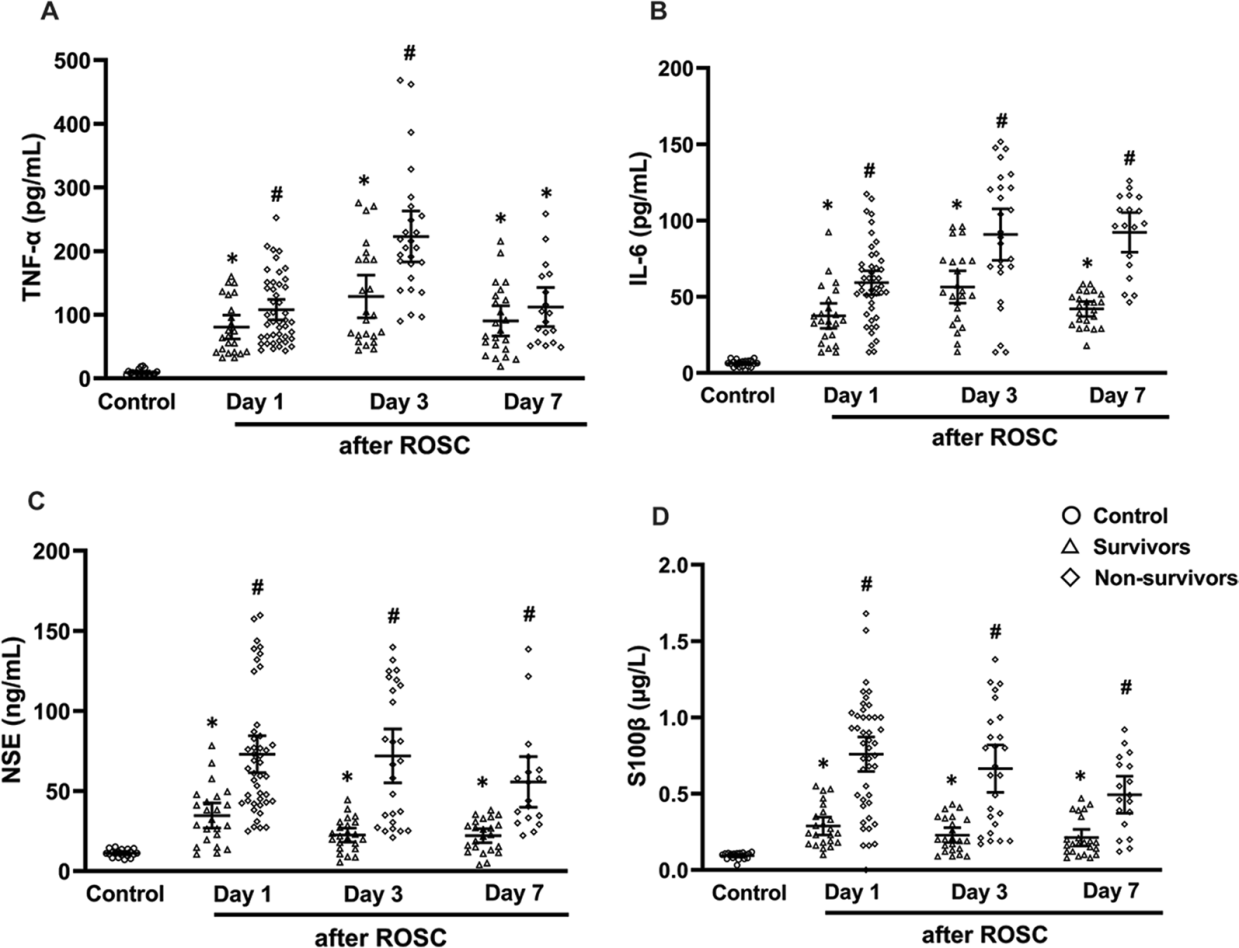
Comparison of serum levels of TNF-α (**A**), IL-6 (**B**), NSE (**C**) and S100β (**D**) in healthy volunteers (control), survivors and non-survivors. IL-6 interleukin-6, NSE neuron-specific enolase, ROSC restoration of spontaneous circulation, S100β soluble protein 100β, TNF-α tumor necrosis factor-α. ^*^P < 0.05 versus healthy volunteers; ^#^*P* < 0.05 versus Survivors

In the first week after ROSC, serum concentrations of TNF-α and IL-6 levels were significantly elevated in patients after ROSC compared to healthy volunteers (both *P* < 0.05, Fig. 5A and B). Moreover, there were higher serum TNF-α levels on day 1 and 3 after ROSC in the non-survivors compared to the survivors (both *P* < 0.05, Fig. 5A). Serum concentrations of IL-6 on day 1, 3 and 7 after ROSC were significantly increased in the non-survivors compared to the survivors (all *P* < 0.05, Fig. 5B).

In the first week after ROSC, serum NSE and S100β concentrations were significantly elevated in patients compared to the healthy volunteers (both *P* < 0.05, Fig. 5C and B). Moreover, the non-survivors had significantly higher serum NSE and S100β levels than the survivors (both *P* < 0.05, Fig. 5C and D).

### Correlations of serum sCD59 level with serum sC5b-9, TNF-α, IL-6, NSE, S100β, APACHE II score and SOFA score after ROSC

Spearman bivariate correlate analyses revealed that serum sCD59 level was positively correlated with serum sC5b-9, TNF-α, IL-6, NSE, S100β, APACHE II score and SOFA score in the first week after ROSC (all *P* < 0.05, Table 2).

**Table 2:** Correlation of serum sCD59 with other variables in patients after ROSC

	Serum sCD59	
	*r*	*P*
Day 1		
sC5b-9	0.418	0.000
TNF-α	0.306	0.011
IL-6	0.371	0.002
NSE	0.303	0.012
S100β	0.338	0.005
APACHE II score	0.425	0.000
SOFA score	0.429	0.000
Day 3		
sC5b-9	0.553	0.000
TNF-α	0.324	0.023
IL-6	0.406	0.004
NSE	0.530	0.000
S100β	0.382	0.007
APACHE II score	0.489	0.000
SOFA score	0.573	0.000
Day 7		
sC5b-9	0.517	0.001
TNF-α	0.314	0.046
IL-6	0.433	0.005
NSE	0.339	0.030
S100β	0.456	0.003
APACHE II score	0.466	0.002
SOFA score	0.509	0.001

*APACHE II* Acute Physiology and Chronic Health Evaluation II, *IL-6* interleukin-6, *NSE* neuron-specific enolase, *ROSC* restoration of spontaneous circulation, *S100β* soluble protein 100β, *sCD59* soluble CD59, *sC5b-9* soluble C5b-9, *SOFA* Sequential Organ Failure Assessment, *TNF-α* tumor necrosis factor-α

### The values of serum sCD59 for predicting poor 28-day neurological prognosis and all-cause mortality in patients after ROSC

**Fig. 6:**
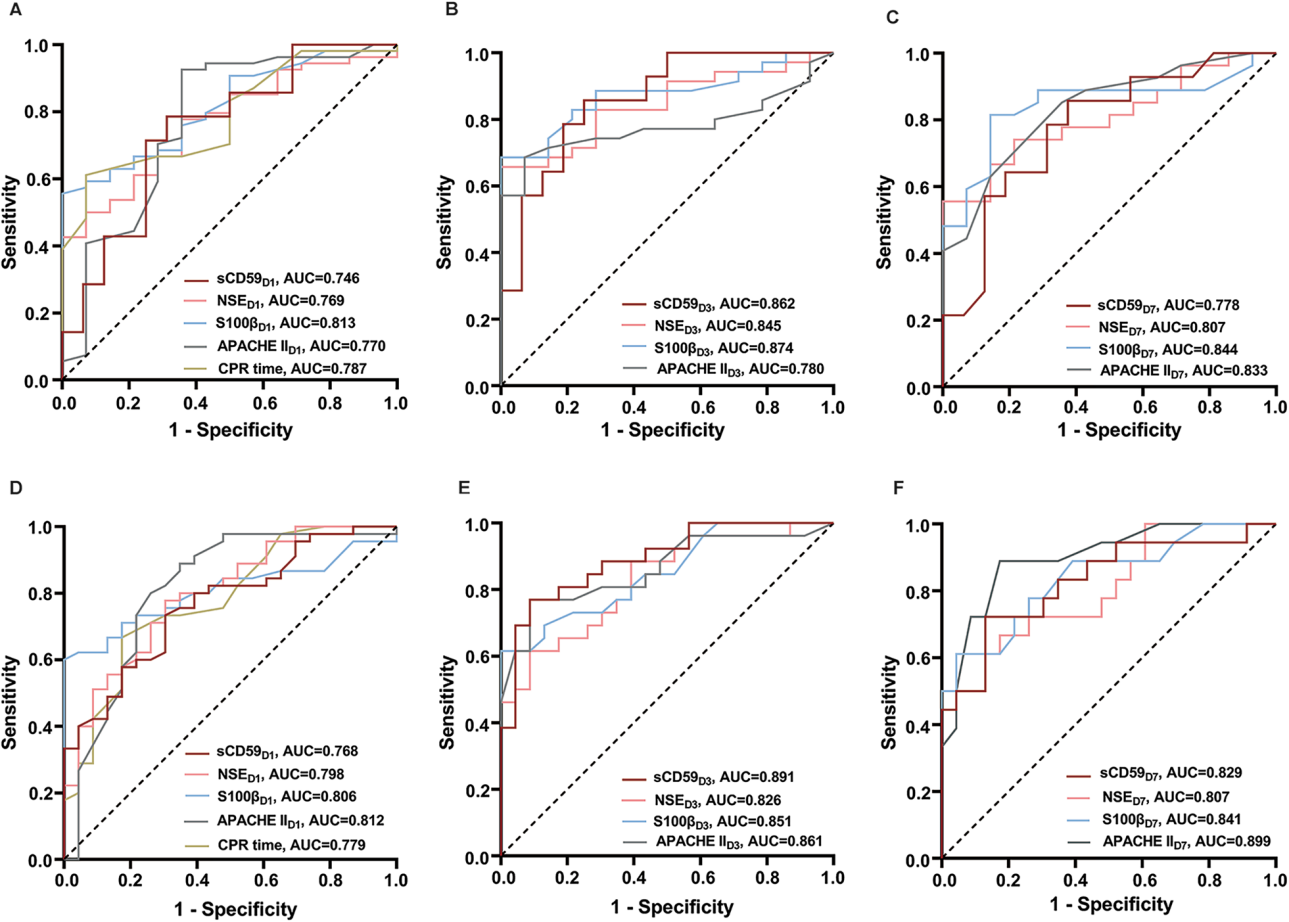
Receiver operating characteristic curves of variables on days 1, 3 and 7 after ROSC for predicting 28-day neurological prognosis (**A**–**C**) and all-cause mortality (**D**–**F**). APACHE Acute Physiology and Chronic Health Evaluation, APACHE II_D1_ APACHE II on day 1, APACHE II_D3_ APACHE II on day 3, APACHE II_D7_ APACHE II on day 7, AUC areas under the curves, CPR cardiopulmonary resuscitation, NSE neuron-specific enolase, NSE_D1_ NSE on day 1, NSE_D3_ NSE on day 3, NSE_D7_ NSE on day 7, ROSC restoration of spontaneous circulation, sCD59 soluble CD59, sCD59_D1_ sCD59 on day 1, sCD59_D3_ sCD59 on day 3, sCD59_D7_ sCD59 on day 7, S100β soluble protein 100β, S100β_D1_ S100β on day 1, S100β_D3_ S100β on day 3, S100β_D7_ S100β on day 7

Serum sCD59, NSE, S100β and APACHE II scores on day 1, 3, and 7, and CPR time had predictive values for poor 28-day neurological prognosis (all *P* < 0.05, Fig. 6A–C, Table 3) and all-cause mortality (all *P* < 0.05, Fig. 6D–F, Table 4). In addition, serum sCD59 level on day 3 after ROSC had the biggest AUC [0.862 (95% CI 0.678–0.960)] for predicting 28-day poor neurological prognosis and the biggest AUC [0.891 (95% CI 0.769–0.962)] for predicting 28-day all-cause mortality (all *P* < 0.05, Fig. 6), together with the highest sensitivity and specificity (Tables 5 and 6).

**Table 3:** Areas under the curve (AUC) of various parameters for predicting poor 28-day neurological prognosis

	AUC	Standard error	*P* value	95% CI
sCD59_D1_	0.746	0.092	0.007	0.554–0.886
sCD59_D3_	0.862	0.067	0.000	0.687–0.960
sCD59_D7_	0.778	0.087	0.001	0.588–0.908
NSE_D1_	0.769	0.063	0.000	0.650–0.862
NSE_D3_	0.845	0.055	0.000	0.713–0.932
NSE_D7_	0.807	0.067	0.000	0.654–0.913
S100β_D1_	0.813	0.055	0.000	0.701– 0.898
S100β_D3_	0.874	0.049	0.000	0.747–0.951
S100β_D7_	0.844	0.064	0.000	0.697–0.938
APACHE II_D1_	0.770	0.083	0.001	0.653–0.864
APACHE II_D3_	0.780	0.065	0.000	0.639–0.886
APACHE II_D7_	0.833	0.064	0.000	0.684–0.931
CPR time	0.787	0.061	0.000	0.671–0.877
sCD59_D1_ + NSE_D1_	0.799^Aa^	0.082	0.005	0.613–0.922
sCD59_D3_ + NSE_D3_	0.955^Bb^	0.034	0.000	0.811–0.997
sCD59_D7_ + NSE_D7_	0.857^Cc^	0.069	0.000	0.681–0.957
sCD59_D1_ + S100β_D1_	0.862^Dd^	0.068	0.000	0.687–0.960
sCD59_D3_ + S100β_D3_	0.960^Ee^	0.030	0.000	0.817–0.998
sCD59_D7_ + S100β_D7_	0.920^Ff^	0.049	0.000	0.761–0.987
sCD59_D1_ + APACHE II_D1_	0.808^Gg^	0.082	0.000	0.623–0.928
sCD59_D3_ + APACHE II_D3_	0.853^Hh^	0.069	0.000	0.676–0.955
sCD59_D7_ + APACHE II_D7_	0.862^Ii^	0.067	0.000	0.687–0.960

^A^ *P* = 0.027 (*Z* = 1.110) vs sCD59_D1_; ^B^ *P* = 0.044 (*Z* = 1.854) vs sCD59_D3_; ^C^ *P* = 0.040 (*Z* = 1.959) vs sCD59_D7_; ^D^ *P* = 0.041 (*Z* = 2.040) vs sCD59_D1_; ^E^ *P* = 0.045 (*Z* = 2.001) vs sCD59_D3_; ^F^ *P* = 0.036 (*Z* = 2.101) vs sCD59_D7_; ^G^ *P* = 0.009 (*Z* = 1.658) vs sCD59_D1_; ^H^ *P* = 0.030 (*Z* = 2.167) vs sCD59_D3_; ^I^ *P* = 0.031 (*Z* = 1.016) vs sCD59_D7_; ^a^ *P* = 0.047 (*Z* = 1.783) vs NSE_D1_; ^b^ *P* = 0.006 (*Z* = 1.857) vs NSE_D3_; ^c^ *P* = 0.047 (*Z* = 2.876) vs NSE_D7_; ^d^ *P* = 0.006 (*Z* = 2.758) vs S100β_D1_; ^e^ *P* = 0.017 (*Z* = 1.752) vs S100β_D3_; ^f^ *P* = 0.013 (*Z* = 2.487) vs S100β_D7_; ^g^ *P* = 0.009 (*Z* = 1.658) vs APACHE II_D1_; ^h^ *P* = 0.030 (*Z* = 2.167) vs APACHE II_D3_; ^i^ *P* = 0.014 (*Z* = 2.455) vs APACHE II_D7_

*APACHE II* Acute Physiology and Chronic Health Evaluation, *APACHE II*_*D1*_ APACHE II on day 1 after ROSC, *APACHE II*_*D3*_ APACHE II on day 3 after ROSC, *APACHE II*_*D7*_ APACHE II on day 7 after ROSC, *CI* confidence interval, *CPR* cardiopulmonary resuscitation, *NSE* neuron-specific enolase, *NSE*_*D1*_ NSE on day 1 after ROSC, *NSE*_*D3*_ NSE on day 3 after ROSC, *NSE*_*D7*_ NSE on day 7 after ROSC, *ROSC* restoration of spontaneous circulation, *sCD59* soluble CD59, *sCD59*_*D1*_ sCD59 on day 1 after ROSC, *sCD59*_*D3*_ sCD59 on day 3 after ROSC, *sCD59*_*D7*_ sCD59 on day 7 after ROSC, *S100β* soluble protein 100β, *S100β*_*D1*_ S100β on day 1 after ROSC, *S100β*_*D3*_ S100β on day 3 after ROSC, *S100β*_*D7*_ S100β on day 7 after ROSC

**Table 4:** Areas under the curve (AUC) of various parameters for predicting 28-day all-cause mortality

	AUC	Standard error	*P* value	95% CI
sCD59_D1_	0.768	0.058	0.000	0.649–0.861
sCD59_D3_	0.891	0.045	0.000	0.769–0.962
sCD59_D7_	0.829	0.068	0.000	0.679–0.928
NSE_D1_	0.798	0.056	0.000	0.683–0.885
NSE_D3_	0.826	0.058	0.000	0.691–0.919
NSE_D7_	0.807	0.069	0.000	0.653–0.913
S100β_D1_	0.806	0.052	0.000	0.693–0.892
S100β_D3_	0.851	0.053	0.000	0.720–0.937
S100β_D7_	0.841	0.064	0.000	0.693–0.936
APACHE II_D1_	0.812	0.062	0.000	0.699–0.897
APACHE II_D3_	0.861	0.054	0.000	0.732–0.943
APACHE II_D7_	0.899	0.049	0.000	0.764–0.971
CPR time	0.779	0.059	0.000	0.662–0.870
sCD59_D1_ + NSE_D1_	0.883^Aa^	0.043	0.000	0.798–0.968
sCD59_D3_ + NSE_D3_	0.937^Bb^	0.034	0.000	0.870–1.000
sCD59_D7_ + NSE_D7_	0.925^Cc^	0.053	0.000	0.820–1.000
sCD59_D1_ + S100β_D1_	0.881^Dd^	0.043	0.000	0.796–0.966
sCD59_D3_ + S100β_D3_	0.983^Ee^	0.013	0.000	0.957–1.000
sCD59_D7_ + S100β_D7_	0.896^Ff^	0.052	0.000	0.794–0.998
sCD59_D1_ + APACHE II_D1_	0.848^G^	0.051	0.000	0.749–0.947
sCD59_D3_ + APACHE II_D3_	0.965^Hh^	0.021	0.000	0.923–1.000
sCD59_D7_ + APACHE II_D7_	0.944^Ii^	0.032	0.000	0.881–1.000

^A^ *P* = 0.023 (*Z* = 2.276) vs sCD59_D1_; ^B^ *P* = 0.022 (*Z* = 2.285) vs sCD59_D3_; ^C^ *P* = 0.041 (*Z* = 1.953) vs sCD59_D7_; ^D^ *P* = 0.002 (*Z* = 2.431) vs sCD59_D1_; ^E^ *P* = 0.023 (*Z* = 2.275) vs sCD59_D3_; ^F^ *P* = 0.045 (*Z* = 1.101) vs sCD59_D7_; ^G^ *P* = 0.031 (*Z* = 1.600) vs sCD59_D1_; ^H^ *P* = 0.026 (*Z* = 2.234) vs sCD59_D3_; ^I^ *P* = 0.046 (*Z* = 1.991) vs sCD59_D7_; ^a^ *P* = 0.045 (*Z* = 1.998) vs NSE_D1_; ^b^ *P* = 0.005 (*Z* = 2.813) vs NSE_D3_; ^c^ *P* = 0.037 (*Z* = 1.809) vs NSE_D7_; ^d^ *P* = 0.046 (*Z* = 1.857) vs S100β_D1_; ^e^ *P* = 0.006 (*Z* = 2.712) vs S100β_D3_; ^f^ *P* = 0.032 (*Z* = 1.199) vs S100β_D7_; ^h^ *P* = 0.026 (*Z* = 2.217) vs APACHE II_D3_; ^i^ *P* = 0.031 (*Z* = 1.442) vs APACHE II_D7_

*APACHE II* Acute Physiology and Chronic Health Evaluation, *APACHE II*_*D1*_ APACHE II on day 1 after ROSC, *APACHE II*_*D3*_ APACHE II on day 3 after ROSC, *APACHE II*_*D7*_ APACHE II on day 7 after ROSC, *CI* confidence interval, *CPR* cardiopulmonary resuscitation, *NSE* neuron-specific enolase, *NSE*_*D1*_ NSE on day 1 after ROSC, *NSE*_*D3*_ NSE on day 3 after ROSC, *NSE*_*D7*_ NSE on day 7 after ROSC, *ROSC* restoration of spontaneous circulation, *sCD59* soluble CD59, *sCD59*_*D1*_ sCD59 on day 1 after ROSC, *sCD59*_*D3*_ sCD59 on day 3 after ROSC, *sCD59*_*D7*_ sCD59 on day 7 after ROSC, *S100β* soluble protein 100β, *S100β*_*D1*_ S100β on day 1 after ROSC, *S100β*_*D3*_ S100β on day 3 after ROSC, *S100β*_*D7*_ S100β on day 7 after ROSC

**Table 5:** Performance of variables for predicting poor 28-day neurological prognosis

	Cut-off	Sensitivity (%)	Specificity (%)	PPV (%)	NPV (%)	Youden (%)	LR+	LR–
sCD59_D1_ (ng/mL)	18.71	78.57	68.75	68.7	78.6	47.3	2.51	0.31
sCD59_D3_ (ng/mL)	23.22	85.71	75.00	75.0	85.7	60.7	3.43	0.19
sCD59_D7_ (ng/mL)	20.31	85.71	62.50	66.7	83.3	48.2	2.29	0.23
NSE_D1_ (ng/mL)	57.71	50.00	92.86	96.4	32.5	42.8	7.00	0.54
NSE_D3_ (ng/mL)	30.63	65.71	92.86	95.8	52.0	65.7	9.20	0.37
NSE_D7_ (ng/mL)	35.41	55.56	85.71	93.8	52.0	55.6	7.78	0.48
S100β_D1_ (μg/L)	0.53	57.41	92.86	96.9	36.1	55.6	8.04	0.46
S100β_D3_ (μg/L)	0.41	68.57	92.86	96.0	54.2	68.6	9.60	0.34
S100β_D7_ (μg/L)	0.21	81.48	85.71	91.7	70.6	67.2	5.70	0.22
APACHE II_D1_	21	92.59	64.29	90.9	69.2	56.9	2.59	0.12
APACHE II_D3_	34	68.57	92.86	96.0	54.2	61.4	9.60	0.34
APACHE II_D7_	25	85.19	64.29	82.1	69.2	49.5	2.39	0.23
CPR time (min)	9	61.11	92.86	97.1	38.2	53.9	8.56	0.42
sCD59_D1_ + NSE_D1_	–	85.71	75.00	75.0	85.7	60.7	3.43	0.19
sCD59_D3_ + NSE_D3_	–	85.71	93.75	92.3	88.2	79.5	13.71	0.15
sCD59_D7_ + NSE_D7_	–	64.29	93.75	90.0	75.0	64.3	10.29	0.38
sCD59_D1_ + S100β_D1_	–	92.86	68.75	72.2	91.7	68.7	2.97	0.10
sCD59_D3_ + S100β_D3_	–	92.86	81.25	81.2	92.9	81.3	4.95	0.09
sCD59_D7_ + S100β_D7_	–	71.43	93.75	90.9	78.9	71.4	11.43	0.30
sCD59_D1_ + APACHE II_D1_	–	78.57	75.00	73.3	80.0	53.6	3.14	0.29
sCD59_D3_ + APACHE II_D3_	–	92.86	68.75	72.2	91.7	68.8	2.97	0.10
sCD59_D7_ + APACHE II_D7_	–	71.43	93.75	90.9	78.9	65.2	11.43	0.30

*APACHE II* Acute Physiology and Chronic Health Evaluation, *APACHE II*_*D1*_ APACHE II on day 1 after ROSC, *APACHE II*_*D3*_ APACHE II on day 3 after ROSC, *APACHE II*_*D7*_ APACHE II on day 7 after ROSC, *CI* confidence interval, *CPR* cardiopulmonary resuscitation, *NSE* neuron-specific enolase, *NSE*_*D1*_ NSE on day 1 after ROSC, *NSE*_*D3*_ NSE on day 3 after ROSC, *NSE*_*D7*_ NSE on day 7 after ROSC, *ROSC* restoration of spontaneous circulation, *sCD59* soluble CD59, *sCD59*_*D1*_ sCD59 on day 1 after ROSC, *sCD59*_*D3*_ sCD59 on day 3 after ROSC, *sCD59*_*D7*_ sCD59 on day 7 after ROSC, *S100β* soluble protein 100β, *S100β*_*D1*_ S100β on day 1 after ROSC, *S100β*_*D3*_ S100β on day 3 after ROSC, *S100β*_*D7*_ S100β on day 7 after ROSC, *LR*+ positive likelihood ratio, *LR–* negative likelihood ratio, *NPV* negative predictive value, *PPV* positive predictive value

**Table 6:** Performance of variables for predicting 28-day all-cause mortality prognosis

	Cut-off	Sensitivity (%)	Specificity (%)	PPV (%)	NPV (%)	Youden (%)	LR+	LR–
sCD59_D1_ (ng/mL)	19.23	73.33	69.57	82.5	57.1	42.9	2.41	0.38
sCD59_D3_ (ng/mL)	26.33	76.92	91.30	90.9	77.8	68.2	8.85	0.25
sCD59_D7_ (ng/mL)	29.45	72.22	86.96	81.2	80.0	59.2	5.54	0.32
NSE_D1_ (ng/mL)	41.26	77.78	69.57	83.3	61.5	47.3	2.56	0.32
NSE_D3_ (ng/mL)	34.28	61.54	91.30	88.9	67.7	52.8	7.08	0.42
NSE_D7_ (ng/mL)	38.10	50.00	91.30	81.8	70.0	50.0	5.75	0.55
S100β_D1_ (μg/L)	0.55	62.22	95.65	96.6	56.4	60.0	14.31	0.39
S100β_D3_ (μg/L)	0.43	61.54	95.65	94.1	68.7	61.5	14.15	0.40
S100β_D7_ (μg/L)	0.40	61.11	91.30	84.6	75.0	56.8	7.03	0.43
APACHE II_D1_	26	88.89	65.22	83.3	75.0	54.1	2.56	0.17
APACHE II_D3_	30	76.92	91.30	90.9	77.8	68.2	8.85	0.25
APACHE II_D7_	24	88.89	82.61	80.0	90.5	71.5	5.11	0.13
CPR time (min)	8	66.67	82.61	88.2	55.9	49.3	3.83	0.40
sCD59_D1_ + NSE_D1_	–	82.22	91.30	94.9	72.4	73.5	9.46	0.19
sCD59_D3_ + NSE_D3_	–	92.31	86.96	88.9	90.9	79.3	7.08	0.09
sCD59_D7_ + NSE_D7_	–	94.44	86.96	85.0	95.2	81.4	7.24	0.06
sCD59_D1_ + S100β_D1_	–	84.44	91.30	95.0	75.0	75.8	9.71	0.17
sCD59_D3_ + S100β_D3_	–	92.31	95.65	96.0	91.7	87.9	21.23	0.08
sCD59_D7_ + S100β_D7_	–	88.89	86.96	84.2	90.9	75.9	6.81	0.13
sCD59_D1_ + APACHE II_D1_	–	97.78	60.87	83.0	93.3	58.7	2.50	0.04
sCD59_D3_ + APACHE II_D3_	–	80.77	100.00	100.0	82.1	80.8	–	0.19
sCD59_D7_ + APACHE II_D7_	–	100.00	78.26	78.3	100.0	78.3	4.60	0.00

*APACHE II* Acute Physiology and Chronic Health Evaluation, *APACHE II*_*D1*_ APACHE II on day 1 after ROSC, APACHE II_D3_ APACHE II on day 3 after ROSC, *APACHE II*_*D7*_ APACHE II on day 7 after ROSC, *CI* confidence interval, *CPR* cardiopulmonary resuscitation, *NSE* neuron-specific enolase, *NSE*_*D1*_ NSE on day 1 after ROSC, *NSE*_*D3*_ NSE on day 3 after ROSC, *NSE*_*D7*_ NSE on day 7 after ROSC, *ROSC* restoration of spontaneous circulation, *sCD59* soluble CD59, *sCD59*_*D1*_ sCD59 on day 1 after ROSC, *sCD59*_*D3*_ sCD59 on day 3 after ROSC, *sCD59*_*D7*_ sCD59 on day 7 after ROSC, *S100β* soluble protein 100β, *S100β*_*D1*_ S100β on day 1 after ROSC, *S100β*_*D3*_ S100β on day 3 after ROSC, *S100β*_*D7*_ S100β on day 7 after ROSC, *LR*+ positive likelihood ratio, *LR–* negative likelihood ratio, *NPV* negative predictive value, *PPV* positive predictive value

**Fig. 7:**
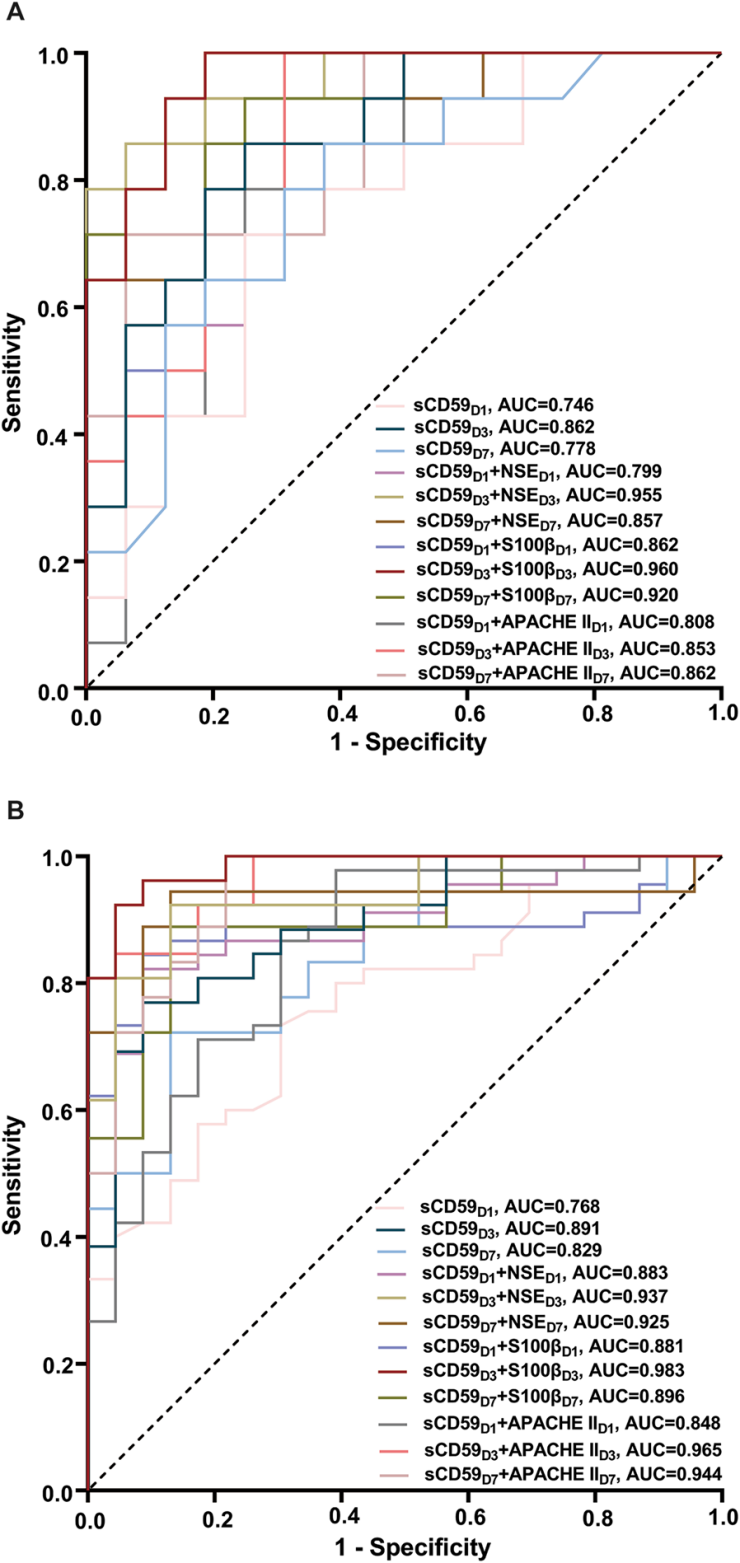
Receiver operating characteristic curves of serum sCD59 and combination with NSE, S100β and APACHE II scores for predicting 28-day neurological prognosis (**A**) and all-cause mortality (**B**). APACHE II Acute Physiology and Chronic Health Evaluation, AUC areas under the curves, NSE neuron-specific enolase, ROSC restoration of spontaneous circulation, sCD59 soluble CD59, S100β soluble protein 100β

We also determined the values of the combination of sCD59 with NSE, S100β and APACHE II scores for predicting 28-day neurological prognosis and all-cause mortality (Fig. 7). AUCs for the combination of serum sCD59 with NSE, S100β and APACHE II scores on days 1, 3 and 7 added the predictive values of serum sCD59, NSE, S100β or APACHE II scores alone (Tables 3 and 4). Tables 5 and 6 present the cut-off value, sensitivity, specificity, PPV, NPV, Youden Index, LR+, and LR− of variables for predicting poor 28-day neurological prognosis and all-cause mortality.

In univariate analysis, variables with *P* values less than 0.1 were available for multivariate analysis. In this study, only age > 65 years, initial cardiac rhythm, CPR time (min), sCD59_D1_ (ng/mL), APACHE II scores and SOFA scores were selected for multivariate binary logistic regression because the sample size was not large enough (Tables 7 and 8). APACHE II and SOFA scores were removed in multivariate binary logistic regression to alleviate the effects of multicollinearity because age, APACH II, and SOFA could be multicollinearity. After adjusting for age, initial cardiac rhythm, and CPR time in the multivariable analysis, sCD59_D1_ remained independently associated with poor 28-day neurological prognosis (OR: 2.435, 95% CI 1.168–5.078, *P* = 0.018) and all-cause mortality (OR: 2.060, 95% CI 1.178–3.604, *P* = 0.011) (Tables 7 and 8).

**Table 7:** Univariate and multivariate logistic regression analyses of poor 28-day neurological prognosis in patients with ROSC

	Univariate analysis			Multivariate analysis		
	OR value	95% CI	*P* value	OR value	95% CI	*P* value
Age > 65 years	3.918	1.140–13.469	0.030	0.636	0.072–5.599	0.683
Initial cardiac rhythm	2.902	0.870–9.676	0.083	1.297	0.149–11.276	0.814
CPR time (min)	1.545	1.197–1.994	0.001	1.500	1.077–2.090	0.016
sCD59_D1_ (ng/mL)	2.767	1.608–4.761	0.000	2.435	1.168–5.078	0.018
APACHE II scores	1.170	1.074–1.275	0.000			
SOFA scores	1.253	1.022–1.536	0.030			

*APACHE II* Acute Physiology and Chronic Health Evaluation II, *CPR* cardiopulmonary resuscitation, *CI* confidence interval, *OR* odds ratio, *sCD59*_*D1*_ sCD59 on day 1 after ROSC, *ROSC* restoration of spontaneous circulation, *SOFA* Sequential Organ Failure Assessment

**Table 8:** Univariate and multivariate logistic regression analyses of 28-day all-cause mortality prognosis in patients with ROSC

	Univariate analysis			Multivariate analysis		
	OR value	95% CI	*P* value	OR value	95% CI	*P* value
Age > 65 years	3.829	1.331–11.016	0.013	3.601	0.754–17.207	0.108
Initial cardiac rhythm	3.111	1.098–8.818	0.033	2.758	0.479–15.878	0.256
CPR time (min)	1.197	1.076–1.331	0.001	1.185	1.035–1.357	0.014
sCD59_D1_ (ng/mL)	2.330	1.500–3.618	0.000	2.060	1.178–3.604	0.011
APACHE II scores	1.176	1.086–1.273	0.000			
SOFA scores	1.181	1.002–1.392	0.048			

*APACHE II* Acute Physiology and Chronic Health Evaluation II, *CPR* cardiopulmonary resuscitation, *CI* confidence interval, *OR* odds ratio, *sCD59*_*D1*_ sCD59 on day 1 after ROSC, *ROSC* restoration of spontaneous circulation, *SOFA* Sequential Organ Failure Assessment

## Discussion

This study indicated that complement activation occurs immediately after ROSC. More importantly, sCD59, as a soluble form of complement regulatory protein CD59 that inhibits MAC formation, was significantly elevated in patients after ROSC, with a significant elevation in the non-survivors compared to survivors. The elevated sCD59 was closely correlated with the elevation of pro-inflammatory mediators and neurological injury biomarkers. Moreover, increased serum sCD59 levels may indicate poor neurological prognosis and survival outcome. Finally, we found that, among the examined variables, increased serum sCD59 had the best performance in predicting the poor 28-day neurological prognosis and all-cause mortality.

Complement activation has been observed during CPR and after ROSC in humans [28]. Here, we measured eight serum complement components (C1q, Bb, MBL, C3b, C3a, C5a, sC5b-9 and sCD59) to determine the extent of complement activation in patients after ROSC. C1q, Bb, MBL and C3b are the markers for the activation of the classic complement pathway, alternative complement pathway, MBL pathway, and converging point of the three pathways, respectively [6]. Our findings showed that the above four complement components were all significantly increased in patients after ROSC compared to healthy volunteers. These findings indicated that these three complement pathways were all activated in patients after ROSC, which is in accordance with a recent study [7]. After complement activation, C3a and C5a, as pro-inflammatory mediators, can contribute to inflammatory cascade, as evidenced by the elevated serum levels of IL-6 and TNF-α after ROSC in this study. In addition, the cytolytic sC5b-9, as the common end-product of the classical, lectin, and alternative complement pathways, can directly damage cell membrane. Therefore, the elevated serum complement activation products, including C3a, C5a, and sC5b-9, have been reported to be associated with post-cardiac arrest immunoinflammatory response and poor prognosis [7, 11, 29], which is consistent with our result that the non-survivors had higher concentrations of serum C3a, C5a and sC5b-9 than the survivors.

Serum sCD59, inhibiting the formation of C5b-9 through preventing the incorporation and polymerization of C9 on cell membranes, was elevated in patients with sepsis, severe acute pancreatitis, acute myocardial infarction, and lung transplantation [19, 27, 30, 31]. The exact mechanisms how CD59 sheds from the cell membrane and is released into the circulation in a soluble form (sCD59) are complex. These mechanisms may be related to enzymatic proteolytic or lipolytic cleavage, or as part of released exosomes [20, 32, 33]. Notably, a study using indirect immunofluorescence microscopy to analyze heart sections showed that substantial C5b-9 deposition may lead to CD59 release [34]. This study aimed to observe the expression of CD59 in human heart and the relationship between C5b-9 deposition and CD59 in myocardial tissue specimens obtained at autopsy from patients who had died of myocardial infarction [34]. In addition, the immunoblotting and immunofluorescence analysis revealed that the CD59 was observed in the sarcolemmal membranes of normal myocardium, but was lost or clearly diminished in infarcted areas during 1–14 days after the onset of myocardial infarction. Especially, loss of CD59 expression was accompanied by concomitant deposition of the C5b-9 within the CD59-negative lesions. In addition, in infarcted border zone, CD59 was often observed in small vesicles, suggesting shedding as a possible mechanism for its release. Therefore, it is understandable that serum sCD59 also is elevated after ROSC as a consequence of whole-body I/R injury, which has been observed in the present study.

As mentioned above, the deposition of C5b-9 as an end-product of complement activation on cell membrane may lead to CD59 release during ischemia in patients with acute myocardial infarction [27, 34]. Therefore, it is reasonable that elevated serum level of sCD59 is positively related to the increased formation of C5b-9 or sC5b-9 during I/R after ROSC, which has also been conformed in the present study. Considering that CD59 is broadly expressed in cells from hemopoietic and non-hemopoietic origin, it can be reasonably speculated that sCD59 may be released from the cells in most organs and tissues, including the cells in brain, as a consequence of the increased formation of C5b-9 caused by systemic I/R after ROSC [18]. Therefore, it is also plausible that the elevated serum sCD59 was positively correlated with APACHE II score, SOFA score and the serum brain injury biomarkers NSE, S100β. The present study also found that serum sCD59 level was higher in the non-survivors than that in the survivors, which might be due to worse I/R-associated inflammation and tissue injury after ROSC [27, 35]. These findings revealed a close relationship between the elevated sCD59 and severe brain injury and poor outcome, which is consistent with the results from patients with sepsis, severe acute pancreatitis, acute myocardial infarction, and lung transplantation [19, 27, 30, 31]. What’s more, we found that serum CD59 also showed decent predictive values for poor 28-day neurological prognosis and all-cause mortality in patients after ROSC. Especially, the combination of serum sCD59 with the two commonly used blood biomarkers NSE and S100β as well as APACHE II scores added the predictive values of serum sCD59, NSE, S100β or APACHE II scores alone.

However, the relationship between the elevated sCD59 and severe brain injury and outcome could be indirect, but not causal. This might be explained by the following reasons. CD59 exerts a protective effect on the complement-induced damage by blocking the formation of C5b-9. In a cerebral I/R model, CD59 deficient mice showed poor outcomes after I/R, suggesting that CD59 protects against ischemic brain damage [36]. Likewise, upregulating the expression of CD59 in neurons protects neurons from complement-mediated damage [37]. In addition, sCD59, as a soluble isoforms of CD59, retains their specific binding activity towards the C5b-9 to exert a protective effect though it only has a limited ability to inhibit C5b-9 assembly on cell membranes spholipid tail [20]. Indeed, the increased C5b-9 is exactly one of the direct causes that lead to severe brain injury and poor outcome. Therefore, we speculated that the close relationship of the elevated sCD59 to severe brain injury and poor outcome could be due to the positive correlation of the elevated sCD59 with C5b-9 formation, as supported by the mechanism that substantial C5b-9 deposition may lead to CD59 release [34].

This study is not without limitations. The current sample size may be not convincing to perform multivariate analysis of high-quality. Hence, more patients need to be enrolled from multiple centers to validate these results. Although the elevated sCD59 level in cerebrospinal fluid could contribute to assessing neurological prognosis, we did not measure sCD59 levels of cerebrospinal fluid in patients due to the difficulty in collecting cerebrospinal fluid [38, 39]. Among the included patients after ROSC, 3 patients with refractory cardiogenic shock withdraw life sustaining therapy (vasopressors) ahead of time according to the request of their relatives and died within 1 h. This may have impact to the outcome of the patients. Moreover, the specificity of sCD59 as a biomarker in predicting neurological prognosis and mortality in patients after ROSC is limited due to the complex mechanisms underlying the post-cardiac arrest syndrome. Finally, the exact molecular mechanisms of sCD59 in alleviating whole-body I/R injury, especially brain injury, remains unclear, and further investigations should be performed.

## Conclusions

Complement system is activated in the first week after ROSC in CA patients. The elevated serum level of sCD59 was positively correlated with disease severity after ROSC. In addition, serum sCD59 could have decent predictive values for the 28-day poor neurological outcome and all-cause mortality in patients after ROSC. The combination of serum sCD59 with NSE, S100β and APACHE II scores added the predictive value of each variable alone.

## Supplementary Information

**Additional file 1: Table S1.** Characteristics of survivors and non-survivors on days 3 and 7 after ROSC. **Table S2.** Comparison of sCD59 levels on days 1, 3 and 7 after ROSC in either survivors or non-survivors. **Table S3.** Comparison of sCD59 levels between survivors and early death patients (died within the first 7 days after ROSC) on days 1 and 3 after ROSC. **Table S4.** Comparison of sCD59 levels between patients with cardiac cause and non-cardiac causes in either survivors or non-survivors on days 1, 3 and 7 after ROSC. **Table S5.** Comparison of sCD59 levels between patients with shockable rhythm and non-shockable rhythm in either survivors or non-survivors on days 1, 3 and 7 after ROSC. **Table S6.** Areas under the curve (AUC) of sCD59 of patients with cardiac/non-cardiac causes and shockable rhythm/non-shockable.

## Data Availability

The datasets generated and analyzed during this study are available from the corresponding author upon reasonable request.

## Abbreviations

APACHE II: Acute Physiology and Chronic Health Evaluation II
AUC: Area under the ROC curve
BNP: Brain natriuretic peptide
CA: Cardiac arrest
CPC: Cerebral performance category
CPR: Cardiopulmonary resuscitation
hs-TnI: High-sensitivity troponin I
ICU: Intensive care unit
IHCA: In-hospital cardiac arrest
IL-6: Interleukin-6
LR+: Positive likelihood ratio
LR−: Negative likelihood ratio
NPV: Negative predictive value
NSE: Neuron-specific enolase
OHCA: Out-of-hospital cardiac arrest
PACS: Post-cardiac arrest syndrome
PCT: Procalcitonin
PPV: Positive predictive value
ROC: Receiver operating characteristic
ROSC: Restoration of spontaneous circulation
S100β: Soluble protein 100β
sCD59: Soluble CD59
sC5b-9: Soluble C5b-9
SOFA: Sequential Organ Failure Assessment
TNF-α: Tumor necrosis factor-α
