# Supplementary material for: Predictive value of soluble CD59 for poor 28-day neurological prognosis and all-cause mortality in patients after cardiopulmonary resuscitation: a prospective observatory study
Source: J Intensive Care. 2023 Feb 2;11:3. doi: 10.1186/s40560-023-00653-8 (PMC9893612; doi:10.1186/s40560-023-00653-8)
Supplement: Supplementary file 1 — Additional file 1: Table S1. Characteristics of survivors and non-survivors on days 3 and 7 after ROSC. Table S2. Comparison of sCD59 levels on days 1, 3 and 7 after ROSC in either survivors or non-survivors. Table S3. Comparison of sCD59 levels between survivors and early death patients (died within the first 7 days after ROSC) on days 1 and 3 after ROSC. Table S4. Comparison of sCD59 levels between patients with cardiac cause and non-cardiac causes in either survivors or non-survivors on days 1, 3 and 7 after ROSC. Table S5. Comparison of sCD59 levels between patients with shockable rhythm and non-shockable rhythm in either survivors or non-survivors on days 1, 3 and 7 after ROSC. Table S6. Areas under the curve (AUC) of sCD59 of patients with cardiac/non-cardiac causes and shockable rhythm/non-shockable. [file 40560_2023_653_MOESM1_ESM.docx]

**Table S1** Characteristics of survivors and non-survivors on days 3 and 7 after ROSC

|  | **Survivors** | **Non-survivors** | ***P* value** |
| --- | --- | --- | --- |
| **Day 3** |  |  |  |
| **Number** | 23 | 26 | — |
| **Age** (years) | 58.5 ± 16.1 | 65.6 ± 9.7 | 0.075 |
| **Male sex** [*n* (%)] | 17 (73.9%) | 15 (57.7%) | 0.367 |
| **SOFA scores** | 5.0 (4.0, 7.5) | 13.0 (9.0, 14.0) | 0.000 |
| **APACHE II scores** | 20.0 (14.0, 38.0) | 33.5 (21.8, 38.0) | 0.002 |
| **Day 7** |  |  |  |
| **Number** | 23 | 18 | — |
| **Age** (years) | 58.5 ± 16.1 | 66.8 ± 13.7 | 0.091 |
| **Male sex** [*n* (%)] | 17 (73.9%) | 10 (55.6%) | 0.322 |
| **SOFA scores** | 5.0 (4.0, 7.5) | 11.5 (8.75, 15.0) | 0.000 |
| **APACHE II scores** | 20.0 (14.0, 38.0) | 28.0 (23.7, 36.0) | 0.025 |

Values are the mean ± standard deviation or median (interquartile range). *APACHE II* Acute Physiology and Chronic Health Evaluation II, *ROSC* restoration of spontaneous circulation, *SOFA* Sequential Organ Failure Assessment

**Table S2** Comparison of sCD59 levels on days 1, 3 and 7 after ROSC in either survivors or non-survivors

|  | **Survivors** | | |  | **Non-survivors** | | |
| --- | --- | --- | --- | --- | --- | --- | --- |
|  | **Day 1** | **Day 3** | **Day 7** |  | **Day 1** | **Day 3** | **Day 7** |
| **Number** | 23 | 23 | 23 |  | 45 | 26 | 18 |
| **sCD59** (ng/mL) | 18.26 ± 2.13 | 22.49 ± 3.47^a^ | 25.91 ± 4.05^ab^ |  | 21.04 ± 2.94 | 29.76 ± 5.27^a^ | 32.37 ± 5.77^ab^ |

Data are presented as the mean ± standard deviation. *ROSC* restoration of spontaneous circulation, *sCD59* soluble CD59

^a^*P* < 0.05 *vs* Day 1, ^b^*P* < 0.05 *vs* Day 3

**Table S3** Comparison of sCD59 levels between survivors and early death patients (died within the first 7 days after ROSC) on days 1 and 3 after ROSC

|  | **Survivors** | **Early death patients** | ***P* value** |
| --- | --- | --- | --- |
| **Day 1** |  |  |  |
| **Number** | 23 | 27 | — |
| **sCD59** (ng/mL) | 18.26 ± 2.13 | 22.08 ± 2.75 | 0.000 |
| **Day 3** |  |  |  |
| **Number** | 23 | 8 | — |
| **sCD59** (ng/mL) | 22.49 ± 3.47 | 30.15 ± 3.59 | 0.000 |

Data are presented as the mean ± standard deviation. *ROSC* restoration of spontaneous circulation, *sCD59* soluble CD59

**Table S4** Comparison of sCD59 levels between patients with cardiac cause and non-cardiac causes in either survivors or non-survivors on days 1, 3 and 7 after ROSC

|  | **Survivors** | |  |  | **Non-survivors** | |  |
| --- | --- | --- | --- | --- | --- | --- | --- |
|  | **Cardiac cause** | **Non-cardiac causes** | ***P* value** |  | **Cardiac cause** | **Non-cardiac causes** | ***P* value** |
| **Day 1** |  |  |  |  |  |  |  |
| **Number** | 11 | 12 |  |  | 20 | 25 |  |
| **sCD59** (ng/mL) | 18.19 ± 2.35 | 18.32 ± 2.01 | 0.887 |  | 21.20 ± 2.66 | 20.91 ± 3.20 | 0.745 |
| **Day 3** |  |  |  |  |  |  |  |
| **Number** | 11 | 12 |  |  | 13 | 13 |  |
| **sCD59** (ng/mL) | 23.63 ± 3.55 | 21.45 ± 3.20 | 0.137 |  | 29.96 ± 5.39 | 29.56 ± 5.35 | 0.851 |
| **Day 7** |  |  |  |  |  |  |  |
| **Number** | 11 | 12 |  |  | 8 | 10 |  |
| **sCD59** (ng/mL) | 26.19 ± 3.92 | 25.65 ± 4.32 | 0.758 |  | 34.14 ± 6.15 | 30.96 ± 5.33 | 0.257 |

Data are presented as the mean ± standard deviation. *ROSC* restoration of spontaneous circulation, *sCD59* soluble CD59

**Table S5** Comparison of sCD59 levels between patients with shockable rhythm and non-shockable rhythm in either survivors or non-survivors on days 1, 3 and 7 after ROSC

|  | **Survivors** | |  |  | **Non-survivors** | |  |
| --- | --- | --- | --- | --- | --- | --- | --- |
|  | **Shockable rhythm** | **Non-shockable rhythm** | ***P* value** |  | **Shockable rhythm** | **Non-shockable rhythm** | ***P* value** |
| **Day 1** |  |  |  |  |  |  |  |
| **Number** | 14 | 9 |  |  | 18 | 27 |  |
| **sCD59** (ng/mL) | 18.38 ± 2.26 | 18.08 ± 2.03 | 0.752 |  | 21.13 ± 2.38 | 20.98 ± 3.31 | 0.867 |
| **Day 3** |  |  |  |  |  |  |  |
| **Number** | 14 | 9 |  |  | 12 | 143 |  |
| **sCD59** (ng/mL) | 23.31 ± 3.72 | 21.21 ± 2.79 | 0.162 |  | 30.20 ± 6.39 | 29.39 ± 4.30 | 0.703 |
| **Day 7** |  |  |  |  |  |  |  |
| **Number** | 14 | 9 |  |  | 9 | 9 |  |
| **sCD59** (ng/mL) | 25.35 ± 4.64 | 26.78 ± 2.93 | 0.420 |  | 35.43 ± 4.96 | 31.54 ± 3.65 | 0.077 |

Data are presented as the mean ± standard deviation. *ROSC* restoration of spontaneous circulation, *sCD59* soluble CD59

**Table S6** Areas under the curve (AUC) of sCD59 of patients with cardiac/non-cardiac causes and shockable rhythm/non-shockable

|  | **AUC** | **Standard error** | ***P* value** | **95% CI** |
| --- | --- | --- | --- | --- |
| **Shockable rhythm/non-shockable** |  |  |  |  |
| **sCD59_D1_** | 0.511 | 0.071 | 0.878 | 0.371–0.650 |
| **sCD59_D3_** | 0.510 | 0.084 | 0.904 | 0.346–0.674 |
| **sCD59_D7_** | 0.501 | 0.093 | 0.989 | 0.318–0.684 |
| **Cardiac/non-cardiac causes** |  |  |  |  |
| **sCD59_D1_** | 0.528 | 0.071 | 0.694 | 0.388–0.667 |
| **sCD59_D3_** | 0.573 | 0.083 | 0.379 | 0.411–0.736 |
| **sCD59_D7_** | 0.537 | 0.093 | 0.685 | 0.355–0.719 |

*CI* confidence interval, *ROSC* restoration of spontaneous circulation, *sCD59* soluble CD59, *sCD59_D1_* sCD59 on day 1 after ROSC, *sCD59_D3_* sCD59 on day 3 after ROSC, *sCD59_D7_* sCD59 on day 7 after ROSC
